# Hollow polymer microcapsule embedded transparent and heat-insulating film†

**DOI:** 10.1039/c8ra00801a

**Published:** 2018-03-06

**Authors:** Chae Bin Kim, Nam-Ho You, Munju Goh

**Affiliations:** Institute of Advanced Composite Materials, Korea Institute of Science and Technology (KIST) Chudong-ro 92, Bongdong-eup Wanju-gun Jeonbuk-do 55324 Korea goh@kist.re.kr

## Abstract

We herein report a facile and scalable approach to manufacturing optically transparent and heat-insulating films by incorporating hollow poly(methyl methacrylate) microcapsules into a transparent polymeric matrix. The microcapsule was prepared *via* emulsion polymerization. The size of the microcapsules could be easily controlled from ∼1 to 3 μm by varying the polymerization time in a narrow size distribution. The microcapsules were then mixed with a UV-curable transparent liquid resin and cured by a subsequent light irradiation. The current approach could enhance the thermal barrier property of the films without a significant reduction in the optical transparency. The solid film possessing 30 wt% microcapsules, for example, exhibited a high visible light transmittance (∼80% as measured by UV-vis spectroscopy) and the thermal conductivity was reduced to 0.06 W mK^−1^, corresponding to 46% of the capsule free film. To quantify and verify this result, theoretical models describing a heat transfer in a hollow microsphere composite were used, and the model showed a good agreement with our experimental observations.

## Abstract 2

Highly transparent, heat-insulating films were manufactured by incorporating hollow poly(methyl methacrylate) microcapsules into a transparent polymeric film.

## Graphical abstract

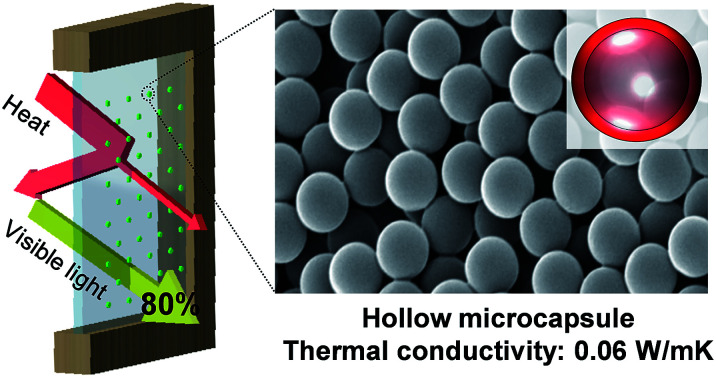

## 1. Introduction

To safely accommodate global increases in population and per capita energy consumption, a significant need for immediate energy conservation has been emphasized all around the world. An effective way to conserve energy is to improve the thermal insulation of buildings. Based on a US Department of Energy (DOE) report, for example, 23% of the electricity used in the US is for the energy supply for space heating, ventilation, and cooling only.^1^ In particular, the energy used to offset unwanted heat losses and gains through windows in buildings was estimated to cost the US ∼$20 billion, which is one-fourth of all the energy used for space heating and cooling.^2^ This is because windows typically occupy ∼20% of the building surface area and it loses more heat in winter and gains more heat in summer than any other surface in the building, ascribing to their high thermal conductivity. Along with the need for thermal insulation, a visible transparency is a prerequisite to the materials used as windows. Therefore, much attention has been focused on improving both the thermal barrier rate and the optical transparency of building windows.^3^

A conventional approach to minimizing the heat loss through windows is placing insulating materials with low thermal conductivities onto or instead of the window glass. Less conductive gas (such as air or argon) filled glazed windows have been widely adapted for the thermal insulation.^4^ However, the process flow including adjustment of the gas mixture securely is somewhat complicated and uneconomical.^5^ Also, the glazed windows may form cracks due to a thermal stress during the usage. As an alternative, silica aerogels have attracted much attention as a thermal insulating material due to its exceptionally high porosity (over 90% pore volume) and low thermal conductivity (∼10 mW mK^−1^).^6–8^ Though wide spread uses of aerogel materials are restricted at present mainly due to their poor mechanical properties, high production costs, and respiratory health issues.^9–11^ Silica aerogel could be somewhat strengthened by combining with polymers, yet the optical transparency usually reduces upon mixing, which greatly limits the applications of the reinforced aerogels as a window insulation film.^12–14^ Many other insulating materials and manufacturing methods including nano-size hollow silicate particles,^15^ nitrogen-doped graphene aerogels,^16^ ceramic fibres/whiskers frameworks,^17^ and urea formaldehyde foam^18^ have been proposed for thermal insulation but usage of them as an insulating window is still limited by optical transparency, complexity of the process, and/or the manufacturing cost.

To this end, we herein describe a facile and scalable approach to manufacturing optically transparent, heat-insulating films by incorporating hollow poly(methyl methacrylate) (PMMA) microcapsules into a transparent polymeric matrix. By incorporating hollow PMMA microcapsules into the transparent films, less thermally conductive air could be efficiently included into the polymeric matrix, thus enhancing the thermal barrier property of the resulting films without a significant reduction in the optical transparency. Using a simple and scalable synthetic route, the hollow PMMA microcapsules were prepared. The capsule sizes were easily tuned by varying the polymerization time in a narrow size distribution. The microcapsules were then mixed with a UV-curable transparent liquid resin and cured by a subsequent light irradiation. Therefore, the current approach could adjust both the optical transparency and the thermal conductivity by simply varying the microcapsule loading.

## 2. Experimental

### Materials

Methyl methacrylate (MMA), dioctylsulfosuccinate sodium salt (AOT), 2,2′-azobisisobutylronitrile (AIBN), poly(vinyl pyrrolidone) (PVP, *M*_n_ ∼ 6 kDa, *Đ* ≤ 1.2), and methanol were purchased from Sigma Aldrich. All other chemicals used in this study were purchased from Fisher Scientific or Sigma-Aldrich, and used as received unless otherwise noted.

### Hollow PMMA microcapsule preparation and characterization

Hollow PMMA microcapsule was prepared following a previously published procedure.^19^ Briefly, AOT (0.9 wt%, 0.45 g), PVP (4 wt%, 4 g), and MeOH (85.45 wt%, 108 mL) were added to the argon purged reactor vessel and allowed to stir for 10 min at room temperature to facilitate the dissolution of AOT and PVP. Then, MMA (10 wt%, 10 mL) and AIBN (0.1 wt%, 0.1 g) were added to the reactor. The reactor was heated to 58 °C and stirred. The weight percentages above are given with respect to the total solution weight. As the reaction proceeded for at least 2 h, the clear solution color turned into translucent solution, indicative of a hollow PMMA microcapsule formation. The obtained PMMA microcapsule was washed with DI-water several times and then freeze dried from water to get fine powder.

Solution state proton nuclear magnetic resonance (^1^H-NMR) measurement was performed using an Agilent NMR 600 MHz spectrometer. Deuterated chloroform (CDCl_3_) was used as the solvent. Microcapsule morphologies were characterized by both polarized optical microscopy (POM, ZEISS, AXIO Imager A2m microscope stand) and scanning electron microscopy (SEM, Nova NanoSEM, FEI, USA) analysis.

### Film preparation and characterization

An UV-curable optically transparent resin (NOA 65) was purchased from Norland Products, Inc. The resin contains a photo-initiator, Irgacure 651. The microcapsule was added into the NOA 65 and allowed to stir for 10 min at room temperature. The percentage of microcapsule added varied from 10–30 wt% with respect to the NOA 65 resin. Films were bar coated onto the poly(ethylene terephthalate) (PET) film with thickness of 100 μm and then UV-cured using a mercury lamp with broadband output from 200–600 nm (Lichtzen UV Cure-60PH) for 2 minutes. The film was dried in vacuum for overnight at room temperature.

UV/vis spectra of films were acquired with a JASCO V-670 UV-Visible Spectrophotometer. Thermal conductivity was measured by the transient plane source method (Hot-disk AB, TPS-2500s).

## 3. Results and discussion

**Fig. 1:**
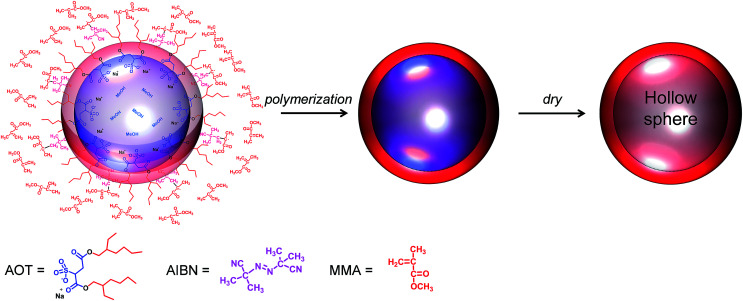
Schematic describing a preparation of the hollow poly(methyl methacrylate) (PMMA) microcapsule.

A schematic describing how the hollow PMMA microcapsules were prepared is shown in Fig. 1. The narrow size distributed, hollow PMMA microcapsules were prepared *via* emulsion polymerization^19^ using 2,2′-azobisisobutylronitrile (AIBN) and methanol as the initiator and solvent, respectively. AIBN, MMA monomer, dioctylsulfosuccinate sodium salt (AOT), poly(vinyl pyrrolidone) (PVP), and methanol were weighed into the reactor. AOT, a surfactant, formed spherical micelles in methanol, templating the amphiphilic interface. After the reactor was purged with nitrogen gas, the polymerization was carried out at 58 °C by stirring at the speed of 400 rpm. Upon heating, the AIBN initiators generated radicals, triggering the polymerization at the amphiphilic interface. In order to inhibit coagulation of the emulsion, PVP was used as a stabilizer.^20^ The obtained transparent PMMA microcapsules were washed with DI-water several times and then freeze dried from water to get fine, hollow microcapsule powder. A proton NMR (^1^H-NMR) analysis of the synthesized PMMA is given as Fig. S1 in ESI.†

**Fig. 2:**
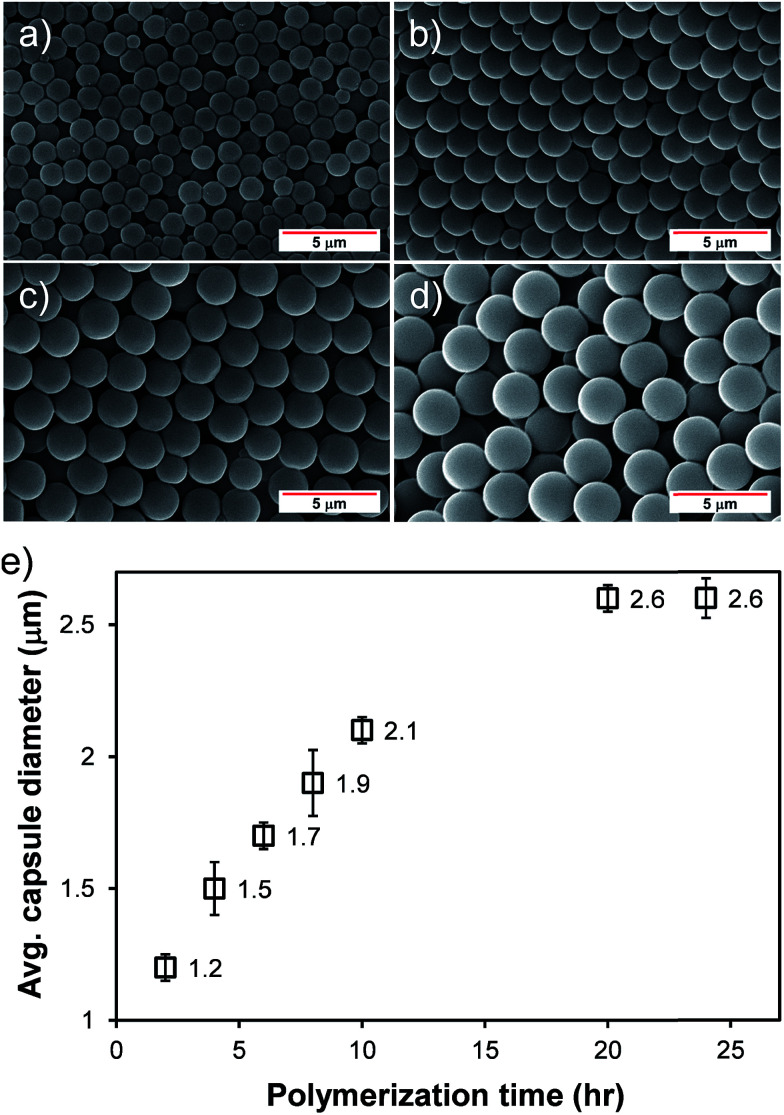
Representative SEM images of the hollow microcapsules after (a) 2 h, (b) 6 h, (c) 10 h, and (d) 20 h of polymerizations. The images were taken at room temperature. The scale bars in this figure correspond to 5 μm. (e) Average microcapsule diameter as a function of polymerization time. The error bars represent the standard deviations of the diameter distributions.

The size of the microcapsule could be controlled in a narrow size distribution by simply varying the polymerization time. As scanning electron micrographs (SEM) shown in Fig. 2a–d display, the microcapsule size increased monotonically with increasing the polymerization time, indicating the PMMA shell gets thicker. After 20 h of the polymerization, the maximum microcapsule size was achieved (∼2.6 μm) and longer polymerization times did not alter the capsule size further. The average capsule diameters shown in Fig. 2e were taken from 30 diameter measurements from the corresponding SEM images. Since the capsule morphologies are dictated by the micelle structures, the capsule morphologies can also be affected in response to changes in other factors such as the surfactant and dispersant types, the amount of surfactants, and *etc.*

**Fig. 3:**
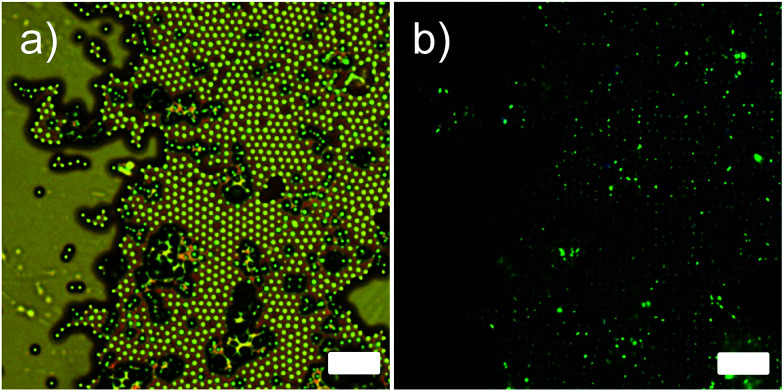
Representative optical micrograph image of liquid crystal (LCs) loaded microcapsule: (a) without a cross-polarizer and (b) with a cross-polarizer. The scale bars in this figure indicate 10 μm.

In order to further confirm the hollow spherical morphology of the microcapsules, microencapsulation experiment using liquid crystals (LCs) was conducted. The synthesized PMMA capsules (0.3 g) were re-dispersed in a mixture of ethanol and 0.25 wt% sodium dodecyl sulfate aqueous solution (in a 1 : 4 volume ratio) by 30 min of sonication. The microcapsules were swollen with an LCs/methylene chloride (0.3/2.7, g/g) emulsion by ultrasonic homogenizing in a 0.25% sodium dodecyl sulfate aqueous solution/ethanol mixture. LCs used were 1 : 1 molar mixture of 4-(*trans*-4-*n*-propylcyclohexyl)ethoxybenzene and 4-(*trans*-4-*n*-propylcyclohexyl) butoxybenzene and they were synthesized following the procedure described elsewhere.^21^ The swelling was carried out by stirring with a magnetic bar at ambient conditions for 3 h until all of the emulsion droplets disappeared completely. The methylene chloride in the swollen PMMA particles was completely evaporated with a rotary evaporator at 30 °C for 1 h. The dispersion was passed repeatedly through a sintered glassing filter (average 2 μm pore diameter) and dried under a vacuum overnight at ambient temperature. Finally, fine LCs-loaded PMMA microcapsules were obtained. Representative optical micrograph images with and without a cross-polarizer on the identical spot of the microcapsule array are shown in Fig. 3. The cross-polarized optical micrograph shown in Fig. 3b clearly displays a birefringence (green color) located only at the center of each capsule, indicating the LCs was successfully encapsulated in the microcapsules. This result reveals that the prepared microcapsules possessed hollow spherical morphology, consistent with the identically prepared microcapsule reported elsewhere.^19,22,23^ This fact also highlights their potential usages in numerous industrial fields including drug delivery, self-healing materials, fragrance release, nutrient preservation, and *etc.*^24–30^

**Fig. 4:**
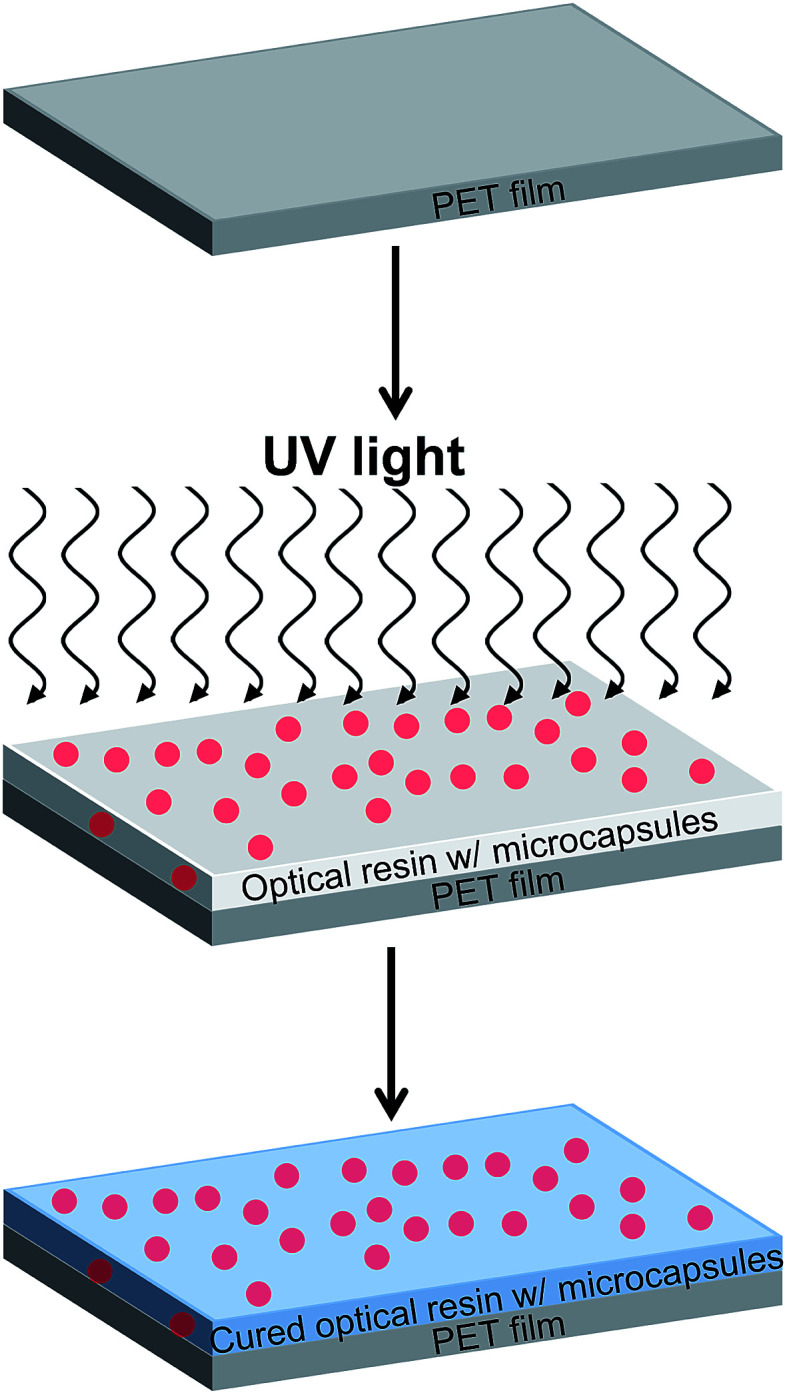
Schematic describing a preparation of the microcapsules embedded transparent film. A UV-curable, transparent liquid resin containing various amounts of microcapsule with a thickness of 80 μm were bar coated onto a 100 μm thick poly(ethylene terephthalate) (PET) substrate and then cured by UV irradiation. The capsule with a diameter of 2.6 μm was used.

As a practical application using the hollow PMMA microcapsules, optically transparent, heat-insulating films were manufactured so that the films can be used as a window insulation film. A schematic describing the process is shown in Fig. 4. Various amounts of the microcapsules were mixed with a UV-curable transparent liquid resin (Norland Optical Adhesive or NOA 65). The percentage of microcapsule was varied from 0–30 wt% with respect to the NOA 65 resin. The microcapsules embedded optical resins were bar coated onto a 100 μm thick poly(ethylene terephthalate) (PET) substrate and then cured with a subsequent light irradiation. The films manufactured by this method, therefore, could include the hollow spherical capsules with their maintained morphologies.

**Fig. 5:**
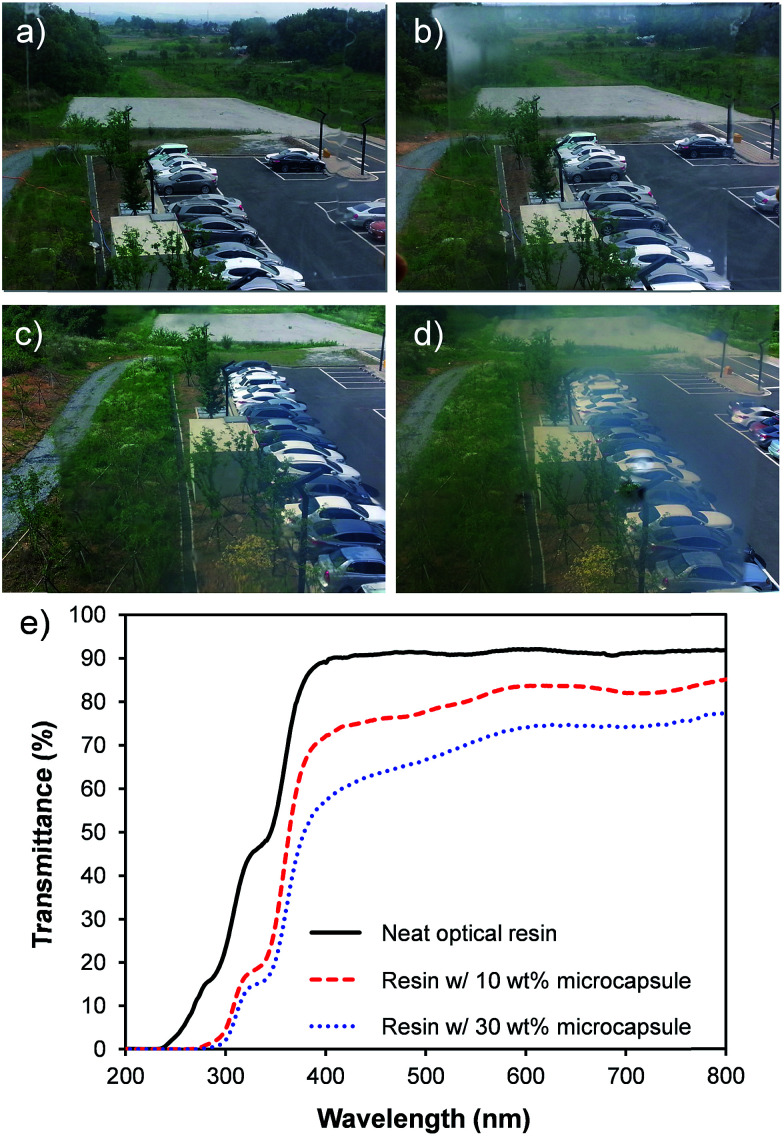
Photograph of the transparent films (80 μm thick) with (a) 0 wt%, (b) 10 wt%, (c) 20 wt%, and (d) 30 wt% hollow microcapsule on a 100 μm thick PET substrate. The weight percentages are given with respect to the matrix weight. (e) UV-vis absorption spectra of the transparent film (80 μm thick) containing 0, 10, and 30 wt% microcapsule on the 100 μm thick PET substrate.

**Fig. 6:**
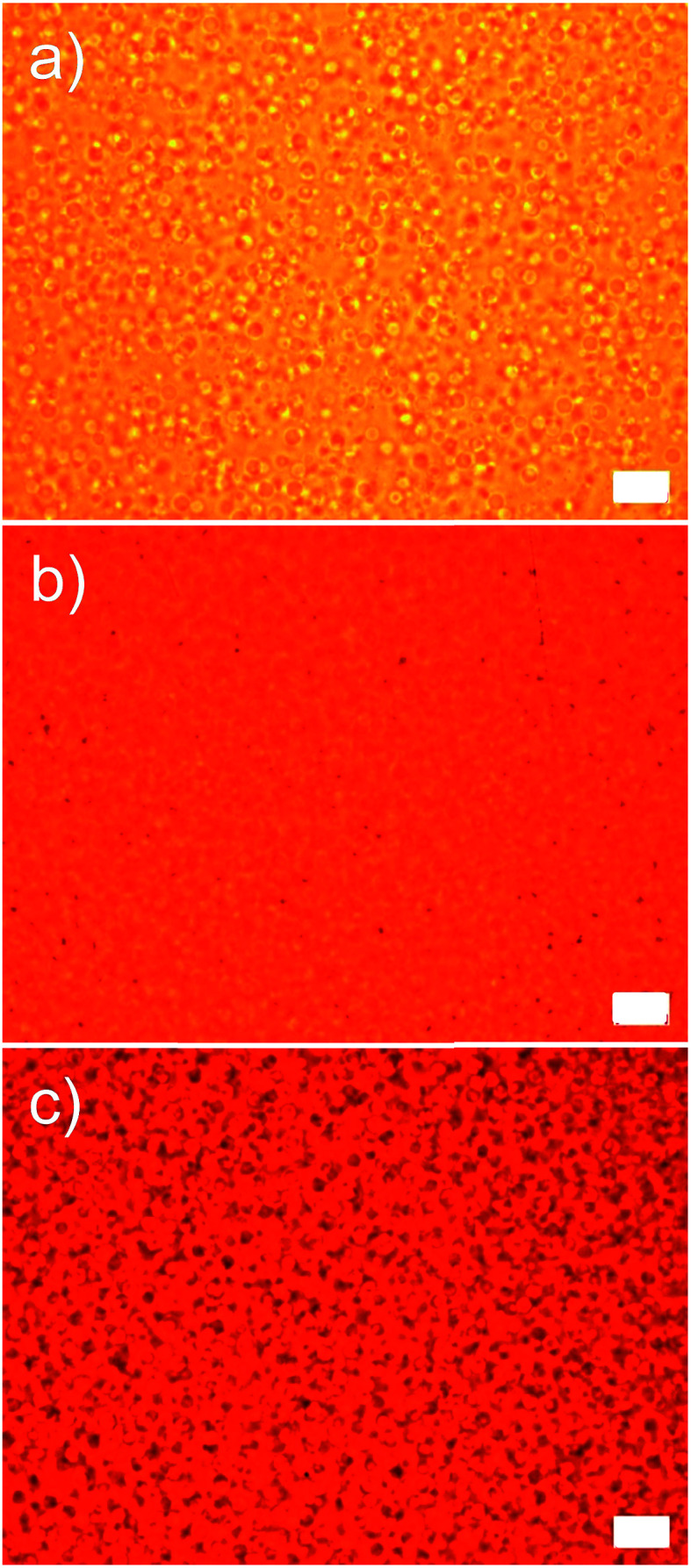
Optical micrographs of the samples possessing (a) 10 wt%, (b) 20 wt%, and (c) 30 wt% microcapsules. All scale bars indicate 10 μm.

A high optical transparency is a crucial aspect for the materials used as a window insulation film. As the representative photographs in Fig. 5a–d display, the films processed as described in Fig. 4 with various amounts of microcapsules up to 30 wt% exhibited a high optical transparency without optical distortions. Accordingly, the films can be directly attached to or used instead of a glass window. All films were 80 μm thick coated on the 100 μm PET substrate. Incorporated microcapsules were well dispersed in the matrix as evidenced by the optical micrographs shown in Fig. 6 and S2 in ESI.† To further demonstrate the optical transparency of the films, UV-vis absorbance spectra were obtained for the films containing 0, 10, and 30 wt% microcapsules and are shown in Fig. 5e. Visible light (a wavelength range from 390 nm to 800 nm) transmittance reduced with increasing microcapsule loadings, attributing to a slight refractive index mismatch. Refractive indices are 1.52, 1.49, and 1.00 for the NOA 65,^31^ PMMA,^32^ and air, respectively. This indicates that higher air content in the microcapsule would result in a lower visible light transmittance. The haze values estimated by UV-vis spectroscopy were 9.9% and 23% for the films containing 10 and 30 wt% microcapsules, respectively. Since the capsule free film on the PET substrate exhibited ∼90% transmittance in a visible light range, the free standing film containing 30 wt% microcapsules (the maximum amount of the capsules used in this study) is anticipated to possess ∼80% visible light transmittance, which the value is higher than those of many commercial window insulation films.^33^

**Fig. 7:**
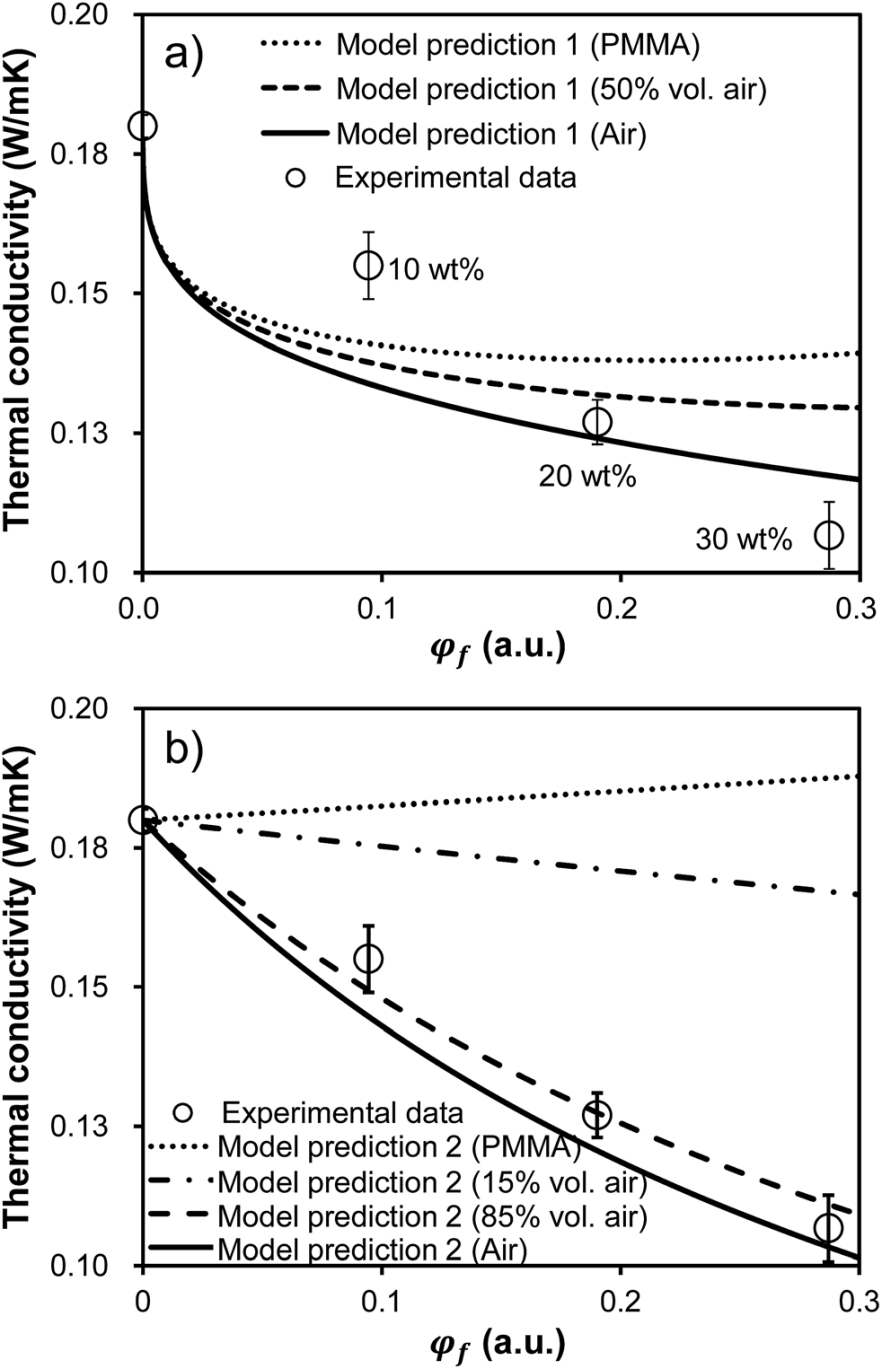
Thermal conductivity of the film as a function of the incorporated microcapsule volume fraction, *φ*_f_. Emptied circles are experimental data. The experimental points in both figures are the identical data. 80 μm thick films were bar coated on the 100 μm thick PET substrates for all samples. Error bars represent standard deviations calculated from three measurements on the identically prepared samples. (a) Three model fits corresponding to the film filled with PMMA microcapsule (*i.e.* no air), half air-filled microcapsule in volume, and pure air sphere (*i.e.* no PMMA) based on eqn (1) are provided. The reported thermal conductivity values in this figure were corrected using eqn (3) in order to account for the sample geometry. (b) Four model fits corresponding to the film filled with PMMA microcapsule (*i.e.* no air), 15 volume% air-filled microcapsule, 85 volume% air-filled microcapsule, and pure air sphere (*i.e.* no PMMA) are provided based on only eqn (3) assuming a parallel multilayered geometry.

In addition to a high optical transparency, low thermal conductivities are also required for the materials to be used as a window insulation film. The transparent films with a thermal conductivity ranging from 0.03 to 0.10 W mK^−1^ are considered as heat insulating films.^34^Fig. 7 shows thermal conductivities of the films as a function of loaded microcapsule volume faction. The thermal conductivities were measured by the transient plane source method (Hot-disk AB, TPS-2500s). An increase in the loaded capsule volume results in a reduction in the thermal conductivities.

Theoretical models would enable efficient optimization of experimental variables to precisely achieve a better material design. Examples of such models include those developed to describe thermal conductivity of hollow glass microsphere and polymer composites.^35–38^ To understand the dependence of the loaded microcapsule volume fraction, *φ*_f_, on thermal conductivities, the experimental results were compared to a theoretical model of heat transfer in hollow microsphere composites, *k*.^39^ The model used in this study is expressed as follow (eqn (1)):1
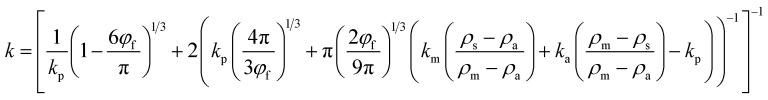
where *k*_p_, *k*_m_, and *k*_a_ are the thermal conductivites of the matrix (optical resin), PMMA shell (0.18 W mK^−1^),^32^ and air (0.025 W mK^−1^) while the *ρ*_m_, *ρ*_a_, and *ρ*_s_ are the densities of the PMMA shell (1180 kg m^−3^),^32^ air (1.226 kg m^−3^), and the mircocapsule. The density of the mircocapsule could be estimated using the eqn (2):

2 *V*_m_*ρ*_m_ + (*V*_s_ − *V*_m_)*ρ*_a_ = *V*_s_*ρ*_s_

where *V*_m_ and *V*_s_ are the volumes of a PMMA shell and the mircocapsule, respectively. The resulting *k* values were then corrected by substituting *k* and a literature value for a thermal conductivity of PET (*k*_PET_; 0.24 W mK^−1^)^32^ into a basic equation of thermal conduction in parallel layers (eqn (3)) with known layer thicknesses in order to account for the sample geometry.3
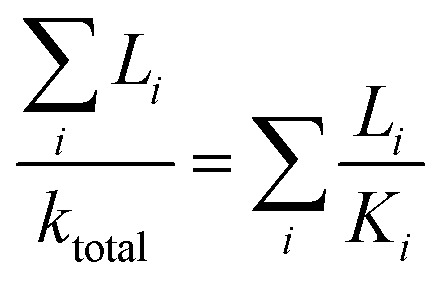


Here, *L* refers to the thickness of layer *i*. By fitting eqn (3) with the experimental value for capsule free film on the PET substrate, *k*_p_ was estimated to be 0.14 W mK^−1^.

Finally, the predicted *k*_total_ along with the experimentally observed values were plotted in Fig. 7a. Three model fits (denoted as Model 1 in Fig. 7a) are provided corresponding to the film filled with PMMA microcapsules (*i.e.* no air), half air-filled microcapsules in volume, and pure air spheres (*i.e.* no PMMA). The model fits predicted the lower thermal conductivities as the loaded microcapsule includes less PMMA (*i.e.* higher air fraction). All model fits exhibited decaying thermal conductivities as a function of the microcapsule loadings, qualitatively agreeing with experimental observations. However, this model effort did not show any quantitative agreement with the experimental data. The experimental values were higher than the predicted values at low capsule loadings, while the model values predicted higher than the experimental values at high capsule loadings. Since the model assumes a system with homogeneously dispersed spheres, sphere aggregation may result in the discrepancy between the model and the experiment.

Since we've confirmed that the incorporated microcapsules were well dispersed in the matrix in in-plane direction by the optical micrograph investigation (see Fig. 6), we postulated that the out-of-plane sphere aggregation could result in the discrepancy between the model and the experiment. The out-of-plane sphere aggregation could occur due to a density difference between the capsule and the liquid matrix during the film fabrication. Accordingly, new model fits for predicting thermal conductivities of the films as a function of *φ*_f_ were given (denoted as Model 2 in Fig. 7b). To simplify the system with the out-of-plane sphere aggregation, this new model assumed a parallel multilayered geometry with four layers including PMMA, air, matrix, and the PET substrate. Therefore, the values were predicted by using only eqn (3). Similar to the results shown in Fig. 7a, the lower conductivities were predicted as increasing the capsule loading and also as increasing the air volume fraction in each capsule. It is noteworthy that a reasonable agreement between data and the model was achieved with 85 volume percent air-filled microcapsules. The quality of agreement obtained with the 85 volume percent air-filled capsules suggests the films in this study possessed highly aggregated capsules in out-of-plane direction rather than well dispersed ones. With the sphere aggregation, the lower thermal conductivities could be achieved than the samples with homogeneously dispersed capsules, implying the films would be more practically beneficial for being used as a window insulation film. Note all reported values in Fig. 7 were taken from the films on the PET substrate with a relatively high thermal conductivity. The thermal conductivity of the free standing film was reduced to 0.06 W mK^−1^ upon incorporating 30 wt% microcapsules (54% reduction compared to the capsule free film), reaching the values suitable for the heat insulating film usage.^34^

## 4. Conclusions

In conclusion, optically transparent, heat insulating films were fabricated by incorporating hollow PMMA microcapsules into a UV-curable, transparent polymeric matrix. Using a simple and scalable synthetic route, the hollow PMMA microcapsules were prepared. The capsule sizes were easily tuned by varying the polymerization time. As the films possessed higher microcapsule contents, the thermal conductivity was monotonically decreased without a significant reduction in the optical transparency. At 30 wt% microcapsule incorporation, the thermal conductivity and the visible light transparency were reduced by 54% and 20%, respectively, compared to the capsule free film. Additionally, models describing heat transfer in hollow microsphere composites were used and compared with our experimental observations. The model effort further revealed the lower thermal conductivity could be achieved with the film possessing out-of-plane hollow sphere aggregation rather than well dispersed ones. In addition to providing fundamental insights, we envision this study could guide future material design in this area.

## Conflicts of interest

There are no conflicts to declare.

## Supplementary Material

RA-008-C8RA00801A-s001
