# Supplementary material for: Hollow polymer microcapsule embedded transparent and heat-insulating film
Source: RSC Adv. 2018 Mar 6;8(17):9480–6. doi: 10.1039/c8ra00801a (PMC9078636; doi:10.1039/c8ra00801a)
Supplement: RA-008-C8RA00801A-s001 [file RA-008-C8RA00801A-s001.pdf]

Electronic Supplementary Material (ESI) for RSC Advances.
This journal is © The Royal Society of Chemistry 2018

Supporting Information for:

Hollow Polymer Microcapsule Embedded Transparent and Heat-insulating Film

*Chae Bin Kim, Nam-Ho, You, and Munju Goh**

Institute of Advanced Composites Materials, Korea Institute of Science and Technology (KIST), Chudong-ro 92, Bongdong-eup, Wanju-gun, Jeonrabuk-do, 55324, Korea

*Corresponding Author. E-mail address: goh@kist.re.kr

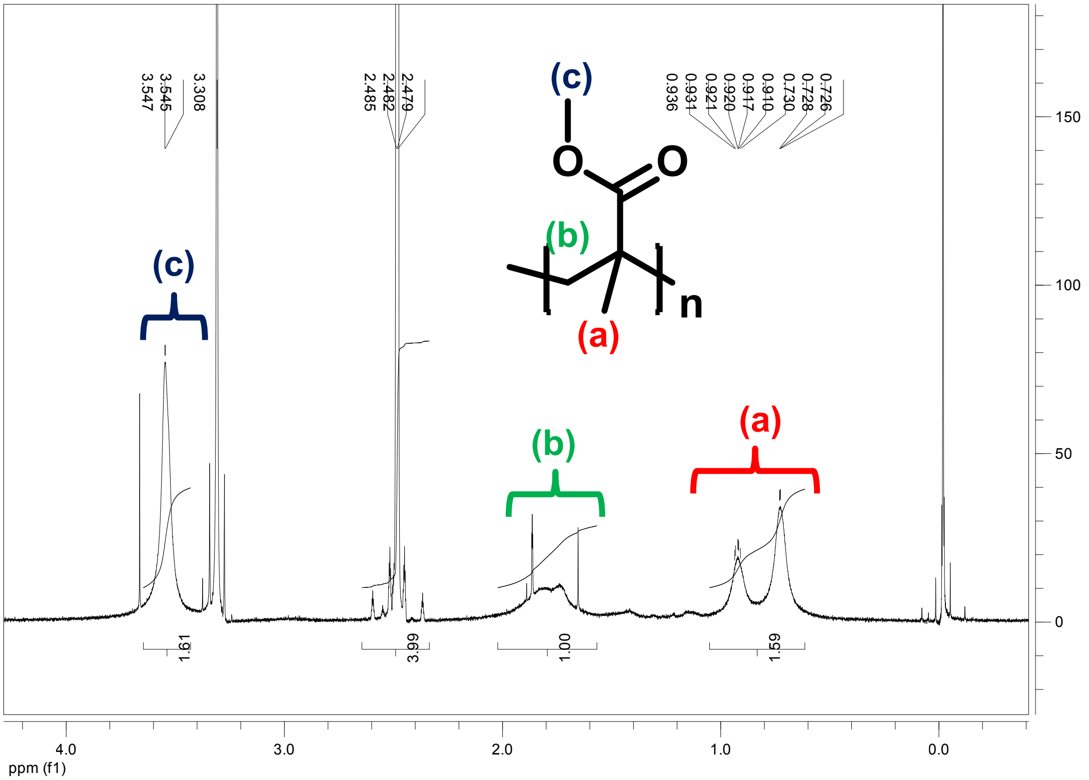

Figure S1. ^{1}H-NMR of the synthesized hollow PMMA microcapsule.

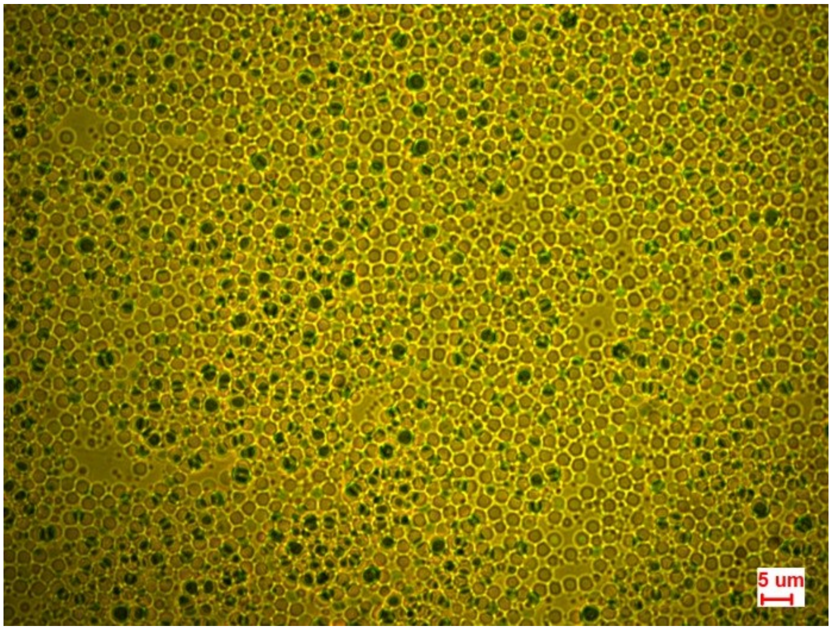

Figure S2. Optical micrograph of the NOA65 sample possessing 30 wt% microcapsules.
